# Excessive nerve growth factor impairs bidirectional communication between the oocyte and cumulus cells resulting in reduced oocyte competence

**DOI:** 10.1186/s12958-018-0349-7

**Published:** 2018-03-27

**Authors:** Yiwen Zhai, Guidong Yao, Faiza Rao, Yong Wang, Xiaoyuan Song, Fei Sun

**Affiliations:** 1 School of Life Sciences, University of Science and Technology of China, Hefei, Anhui People’s Republic of China; 2 Hefei National Laboratory for Physical Sciences at Microscale, Hefei, Anhui People’s Republic of China; 3 Reproductive Medical Center, The First Affiliated Hospital of Zhengzhou University, Zhengzhou, Henan People’s Republic of China

**Keywords:** Polycystic ovary syndrome, NGF, Oocyte-derived paracrine factors, Bidirectional communication, Glycolysis

## Abstract

**Background:**

Excessive nerve growth factor (NGF) is commonly found in the follicular fluid of patients with polycystic ovary syndrome (PCOS). Furthermore, oocytes from PCOS patients exhibit lower developmental competence. The purpose of this study was to explore the association between excessive NGF and low oocyte competence in vitro.

**Methods:**

Excessive NGF was added to mouse cumulus oocyte complexes (COCs) cultured in vitro to investigate meiotic maturation of the oocyte. After culture, mRNA expression levels of *Pfkp* and *Ldha* genes in cumulus cells (CCs) and *Gdf9*, *Bmp15* and *Fgf8* genes in oocytes, were determined by real-time quantitative polymerase chain reaction (qPCR). We also investigated the mRNA content of *Pfkp* and *Ldha* in CCs from PCOS and non-PCOS patients.

**Results:**

Excessive NGF significantly inhibited oocyte meiotic maturation. The inhibitory effect was mediated by the NGF high-affinity receptor, NTRK1. mRNA content of *Pfkp* and *Ldha* genes in CCs was significantly reduced by excessive NGF stimulation. Moreover, the expression levels of *Gdf9*, *Bmp15* and *Fgf8* were also decreased in oocytes, and was induced by excessive NGF-stimulated CCs. In addition, lower expression levels of *Pfkp* and *Ldha* in CCs were identified in Chinese PCOS patients with excessive NGF (PCOS, 22 ± 2.63 ng/ml, *n* = 13; non-PCOS, 7.18 ± 2.42 ng/ml, *n* = 9; *p* < 0.01) in the follicular fluid, suggesting a potential association between excessive NGF and decreased glycolysis in the CCs of women with PCOS.

**Conclusions:**

Excessive NGF impairs bidirectional communication between oocyte and cumulus cells, which might be related to low oocyte competence.

## Background

Nerve growth factor (NGF) is a member of the neurotrophin family [1]. While originally reported to exert functionality in the nervous system, NGF has been found to play important roles in the reproductive system [1, 2]. Various types of cells in the mammalian ovary, including theca cells, granulosa cells and cumulus cells (CCs) have been reported to produce NGF [1, 3]. Consistent with its local production in the ovary, NGF is also known to participate in the regulation of folliculogenesis and ovulation [1, 2, 4–6]. The deficiency of NGF in mice is known to lead to a significant reduction of primary and secondary follicles, implying that NGF is necessary for the preantral-follicle stage [2, 4]. Furthermore, the preovulatory increase of NGF in the follicular fluid of rats, golden hamsters and sheep indicated the involvement of NGF in ovulation [1, 5]. Other studies reported that single nucleotide polymorphism of the NGF gene in goats might be associated with litter size, which also emphasized the importance of NGF in reproduction [7, 8]. NGF has also been reported to play roles in oocyte maturation [9]. The addition of NGF to the in vitro medium promoted the resumption of meiosis in murine oocytes [10], and improves cleavage rate in sheep embryos [11]. However, NGF failed to enhance oocyte maturation and developmental competence in pigs and cows [9, 12]. Thus, the function of NGF in the process of oocyte maturation remains rather controversial and is in need of further elucidation.

Interestingly, an increasing number of studies are providing evidence to show that NGF is associated with ovarian diseases, such as polycystic ovary syndrome (PCOS) [1, 13–15]. PCOS, heterogeneously characterized by polycystic ovaries, hyper-androgenism and chronic anovulation, is a common ovarian disease which causes infertility or sub-fertility in 6–8% of women in reproductive age [16]. The presence of excessive NGF in follicular fluid is a clinical manifestation of PCOS patients [13, 15, 17]. Research has shown that transgenic mice expressing excessive NGF exhibit PCOS-like symptoms, such as the formation of follicular cysts, hyper-androgenemia, increased granulosa cell apoptosis, reduced ovulation and fertility and perturbed reproductive and metabolic features [13, 14]. Nevertheless, the mechanism underlying excessive NGF in the follicular fluid of patients with progressive PCOS remains unclear.

PCOS patients consistently suffer from the poor quality of oocytes [16, 18, 19] and this represents a major cause of sub-fertility or infertility [16, 18, 20]. Although a series of extra- and intra-ovarian factors have been identified and linked to abnormal oocyte maturation [16], the underlying mechanisms still require further clarification. Bidirectional communication between oocytes and the surrounding CCs plays an essential role in oocyte maturation [21–24]. During maturation, oocytes are deficient in synthesizing glucose metabolic enzymes; they use pyruvate as an energy substrate, which is transported from companion CCs [25–27]. Glycolysis in the CCs is dominated by oocyte-derived paracrine factors (ODPFs) [21], of which two families have been identified: the transforming growth factor β superfamily, including growth differentiation factor 9 (GDF9) and bone morphogenetic protein 15 (BMP15); and the fibroblast growth factor (FGF) family, including FGF8 and other members [28–30]. Deficiency of these genes in vivo, such as in *Gdf9*^*−/−*^ and *Gdf9*^*−/−*^*Bmp15*^*−/−*^mice, leads to reproductive defects [23]. The addition of BMP15 and FGF8 to in vitro cultured CCs has been shown to promote glycolysis [31]. Despite the fact that bidirectional communication is important to oocyte maturation, its role in the pathogenesis of PCOS remains undefined.

In the present study, we aimed to clarify the role of excessive NGF in the follicular fluid of PCOS patients and investigated the effect of excessive NGF on oocyte maturation in the mouse model. To mimic excessive NGF in the follicular fluid and dissect it from other micro-environmental factors, we performed in vitro culture of isolated mouse COCs in the presence of excessive NGF. We found that excessive NGF inhibited oocyte maturation in mouse COCs, and that the inhibitory effect was mediated by the bidirectional communication between oocyte and CCs. Moreover, corresponding to the observation that mRNA expression of glycolytic enzymes reduced in mouse CCs when COCs were cultured with excessive NGF, mRNA expression of glycolytic enzymes significantly reduced in CCs isolated from PCOS patients with excessive NGF in the follicular fluid. These findings revealed a potential pathogenic role of excessive NGF in the progression of PCOS by impairing oocyte maturation.

## Methods

### Animals

ICR mice were bred and raised in the Animal Center, University of Science and Technology of China. Mice were adapted to a 12-h light/12-h dark cycle at room temperature (22–24 °C). All experiments involved 21-day-old female mice. The study was approved by the Institutional Review Board of the University of Science and Technology of China on the 18th May 2012 (Reference: USTCACUC1201054).

### Subjects

Follicular fluid and CCs were collected from 22 Han Chinese women (*n* = 9, non-PCOS; *n* = 13, PCOS) during their first cycle of in vitro fertilization (IVF) or intracytoplasmic sperm injection (ICSI) at the First Affiliated Hospital of Zhengzhou University from January 2015 to May 2015. Written informed consent was obtained from all patients and the study was approved by the Ethics Review Board of the First Affiliated Hospital of Zhengzhou University on 20^th^November 2014 (Reference: 2014-LW-1217). All of the PCOS patients were selected according to the Revised 2003 Consensus on Diagnostic Criteria: Oligo- and/or Anovulation, Hyper-androgenism and Polycystic Ovary [32]. All of the non-PCOS patients had normal ovarian morphology and regular menstrual cycles with male factor infertility. Age, ratio of body weight/height, luteinizing hormone (LH), follicle stimulating hormone (FSH) and testosterone levels of both of PCOS patients and non-PCOS patients were measured and recorded.

### Human follicular fluid and CCs collection

After ovarian stimulation with agonadotropin-releasing hormone agonist (Serono, Geneva, Switzerland) and recombinant FSH (Serono, Geneva, Switzerland), three or more follicles reached 17mmin diameter, and then 6000–10,000 IU of human chorionic gonadotropin (hCG, LizhuInc., Zhuhai, China) was administered. COCs were then retrieved from aspirated follicles 36 h after the hCG trigger. Follicular fluid was obtained from the first aspirated follicles and centrifuged at 450 *g* for 5 min; the clear supernatant was then stored at − 80 °C for subsequent NGF assessment. CCs were collected from 9 non-PCOS patients and 5 PCOS patients who received ICSI-embryo transfer, but not IVF-embryo transfer treatment as CCs from such patients would be contaminated with human sperm.

### Measurement of NGF in human follicular fluid

Follicular fluid was first treated with acid as described previously [17, 33] to increase measurable NGF levels. In brief, all samples were diluted 1:15,000 in Dulbecco’s phosphate-buffered saline (DPBS) and treated with 1 mol/L of HCl at pH 2.6 for 30 min. This was then neutralized by 1 mol/L of NaOH to pH 7.6. NGF was then measured using a human β-NGF Duoset ELISA Kit (R&D Systems, Minneapolis, USA) in accordance with the manufacturer’s instructions. In brief, 96-well plates were coated with anti-NGF (human) monoclonal antibody and incubated overnight at 4 °C. All washing steps were conducted 4 times with wash buffer (360 μl/well). Assay plates were washed as above and incubated with blocking buffer for 2 h at room temperature to block non-specific binding. Follicular fluid samples (200 μl/well) and gradient standard NGF samples were added to the appropriate assay wells in duplicate and then incubated for 6 h at room temperature. After incubation, the plates were washed and then detection antibody was added and incubated for 2 h at room temperature. Afterwards, plates were washed and labeled with streptavidin-HRP for 20 min in the dark. Then, substrate solution and stop solution were added into the plates for absorbance measurement. The concentration follicular fluid was calculated using a standard curve.

### Reagents and medium for cell culture

Mouse β-NGF (R&D Systems, Minneapolis, USA) and K252α (Sigma-Aldrich, St. Louis, USA) were prepared at a concentration of 100 ng/ml and 10 mM, as described previously [34]. We used α-MEM culture media (Gibco, Calsbad, USA) supplemented with 0.3% (wt/vol) bovine serum albumen (BSA, Sigma-Aldrich, St. Louis, USA), 0.23 mM pyruvate (Sigma-Aldrich, St. Louis, USA), 75 mg/L penicillin G and 50 mg/L streptomycin sulfate (Gibco, Calsbad, USA).

### Isolation and collection of COCs, oocytectomized cumulus cells (OOX-cumulus cells) and denuded oocytes

Follicle development was stimulated by an intra-peritoneal injection of 5 IU pregnant mare serum gonadotropin (PMSG, Sigma-Aldrich, St. Louis, USA) 44–48 h before harvesting ovaries. Ovaries were removed and large antral follicles were punctured with fine hypodermic needles (number 5 grade) under a dissecting microscope. COCs and denuded oocytes were collected using a mouth-controlled, small-bore glass pipette. OOX-cumulus cells were generated by micro-surgically removing oocytes from the COCs, but leaving the zonapellucida intact, as previously described [23]. In brief, each COC was held by negative pressure with a holding pipette, and most, or all of the oocyte, was removed by negative pressure when a lancing pipette was pushed through the COC. This lead to a spherical zonapellucida and the surrounding CCs are referred to as OOX-cumulus cells hereafter.

During the isolation progress, 10 mM milrinone (Sigma-Aldrich, St. Louis, USA) was used to maintain oocytes at the germinal vesicle (GV) stage. After collection, COCs, OOX-cumulus cells and denuded oocytes were washed in culture medium three times in order to remove milrinone. These cells were then ready for further study.

### Cell culture and evaluation of oocyte meiotic maturation in vitro

COCs were plated in droplets at a density of 1 COC/μl [23, 35]. COCs were then cultured in medium with NGF gradients (1, 10, 100 and 1000 ng/ml) for 18 h, or in medium containing100ng/ml of NGF for 10, 12, and 18 h with or without 10 μM K252α. The proportion of first polar body (PB1) was calculated by removing CCs under a stereomicroscope and evaluating the status of oocyte meiotic maturation.

COCs, OOX-cumulus cells, denuded oocytes and OOX-cumulus cells co-cultured with denuded oocytes [31, 36] (referred as “OOX + DO”) were cultured separately with or without NGF for 18 h, then CCs and oocytes were collected for target gene analysis.

To examine whether NGF-treated CCs inhibit oocyte meiotic maturation, OOX-cumulus cells were first stimulated with NGF for 24 h (1 or 2 OOX-cumulus cells per microliter of medium) and then removed [37]. Denuded oocytes were then added into the supernatants at a concentration of 1 DO/μl for 18 h and then collected for target gene analysis.

### Immunofluorescence

Ovaries were harvested as described above and 20-μm cryosections were prepared and blocked with 5% BSA for 1 h, incubated with rabbit anti-neurotrophic receptor tyrosine kinase 1 (NTRK1) monoclonal antibody (Abcam, Cambridge, UK) overnight at 4 °C, and then labeled with Alexa Fluor 549 donkey anti-rabbit antibody (Jackson Immuno Research, West Grove, USA). Sections were then stained with DAPI (Solarbio, Beijing, China) before mounting with Gold Anti-fade Reagent (Invitrogen, Calsbad, USA). Images were then acquired with an LSM710 (Carl Zeiss, Milano, Italy).

### Real-time qPCR

RNA was extracted using the Dynabeads mRNA DIRECT micro kit (Ambion, Carlsbad, USA). Real-time qPCR was performed using the PrimeScript RT reagent kit (Takara, Shiga, Japan) and the SYBR Premix Ex Taq II Kit (Takara, Shiga, Japan). Primers used for real-time qPCR were selected according to previous reports [31, 34, 38] and listed in Table 1. The specificity of these primers were validated by dissociation curve analysis. Target genes were normalized to *actin* gene. Data were analyzed using the comparative 2^-△△Ct^ method [34].

**Table 1:** Primers for real-time qPCR

Gene name	GenBank accession number	Sequences of forward (F) and reverse (R) primers	Amplification size (bp)
*m-Pfkp*	NM_019703	F: 5′-GCCGTGAAACTCCGAGGAA-3′ R: 5′-GTTGCTCTTGACAATCTTCTCATCAG-3′	96
*m-Ldha*	NM_001136069.2	F: 5′-TGTGGCAGACTTGGCTGAGA-3′ R: 5′-CTGAGGAAGACATCCTCATTGATTC-3′	105
*m-Gdf9*	NM_008110.2	F: 5′-TCACCTCTACAATACCGTCCGG-3′ R: 5′-GAGCAAGTGTTCCATGGCAGTC-3′	139
*m-Bmp15*	NM_009757.4	F: 5′-GCACGATTGGAGCGAAAATG-3′ R: 5′-CGTACGCTACCTGGTTTGATGC-3′	123
*m-Fgf8*	NM_010205.2	F: 5′-CAGGTCTCTACATCTGCATGAACAA-3′ R: 5′-TCTCCAGCACGATCTCTGTGAATA-3′	96
*m-Actin*	NM_007393.5	F:5′-TGGCTCCTAGCACCATGAA-3′ R: 5′-CTCAGTAACAGTCCGCCTAGAAGCA-3′	186
*h-PFKP*	NM_002627.4	F: 5′-AGGCGATGGACGAGAGGAGAT-3′ R: 5′-TGATGGCAAGTCGCTTGTAG-3′	93
*h-LDHA*	NM_001165414.1	F: 5′-TGCACCCAGATTTAGGGACTGAT-3′ R: 5′-CCCAGGATGTGTAGCCTTTGAG-3′	111
*h-ACTIN*	NM_001101.3	F: 5′-TGGCACCCAGCACAATGAA-3′ R: 5′-CTAAGTCATAGTCCGCCTAGAAGCA-3′	186

*m-* mouse, *h-* human, *bp* base pair

### Statistical analysis

Statistical analysis was performed using the Student’s *t-*test. *P* < 0.05 was considered to be statistically significant (*P < 0.05; **P < 001; ***P < 0.001).

## Results

### Excessive NGF is present in the follicular fluid of PCOS patients and inhibits oocyte maturation in vitro

**Fig. 1:**
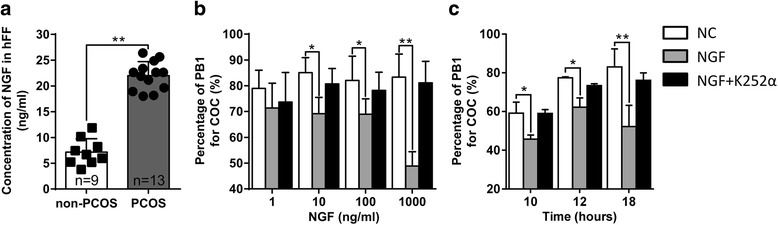
Excessive NGF inhibited mouse oocyte maturation. **a** Excessive NGF in the follicular fluid of Chinese PCOS patients. Concentration of NGF in human follicular fluid (hFF) of Chinese non-PCOS patients (*n* = 9) and PCOS patients (*n* = 13) was determined by ELISA. **b–c** COCs were cultured with the indicated concentration of NGF for 18 h (**b**) or with 100 ng/ml NGF for the indicated times (**c**) in the presence or absence of 10 μM K252α. The proportion of PB1 extrusion was then calculated. The number of COCs for each group was ≥35. Representative data are presented as mean ± SEM. *, *p* < 0.05; **, *p* < 0.01

We firstly detected NGF content in follicular fluid of Chinese PCOS patients. The clinical characteristics of PCOS and non-PCOS patients were shown in Table 2. The concentration of NGF in follicular fluid was significantly higher in the PCOS group (22 ± 2.63 ng/ml, *n* = 13) than that in the non-PCOS group (7.18 ± 2.42 ng/ml, *n* = 9) (Fig. 1a).

**Table 2:** Clinical characteristics of PCOS and non-PCOS patients

	PCOS	non-PCOS	*P* value
Total (*n*)	9	13	
IVF-ET (*n*)	4	4	
ICSI-ET (*n*)	5	9	
Age (year)	29.53 ± 5.61	28.22 ± 2.42	0.46
BMI	24.36 ± 3.24	22.63 ± 2.46	< 0.001
LH (IU/ml)	10.47 ± 4.62	6.23 ± 3.29	< 0.0001
FSH (IU/ml)	5.27 ± 1.41	7.58 ± 2.13	< 0.0001
Testosterone (ng/dl)	52.62 ± 18.74	26.37 ± 11.92	< 0.0001
Antral follicle count (*n*)	34.51 ± 13.08	16.24 ± 5.36	< 0.0001
Starting Gn dose (IU)	152.81 ± 32.24	178.77 ± 36.69	0.0013
Gn stimulation duration (day)	12.43 ± 3.26	10.37 ± 2.17	0.0026
Total Gn administrated (IU)	1899.43 ± 579.52	1933.46 ± 627.41	0.54
E2 level on hCG day (pg/ml)	5768.27 ± 2843.32	4689.66 ± 1895.24	0.005
Follicles ≥14 mm (*n*)	16.43 ± 4.15	12.46 ± 3.53	< 0.0001
Oocytes retrieved (*n*)	18.62 ± 8.05	13.88 ± 4.95	< 0.0001

To observe the effect of NGF on oocyte maturation, mouse COCs from antral follicles were cultured with different concentrations of NGF. This showed that 10 and 100 ng/ml of NGF significantly suppressed PB1 extrusion, a hallmark of oocyte meiotic maturation, and that 1000 ng/ml of NGF had a stronger suppressive effect (Fig. 1b). Taking an overall consideration of both NGF concentrations in the follicular fluid of PCOS patients and their inhibitory effect in vitro, we chose 100 ng/ml as an excessive concentration of NGF that mimics excessive levels of NGF in the follicular fluid of PCOS patients and used this concentration in our subsequent experiments. The inhibitory effect of 100 ng/ml NGF on PB1 extrusion was observed at 10, 12, and 18 h after NGF treatment (Fig. 1c), indicating that the inhibitory effect existed throughout the progression of meiosis I.

### NTRK1, the high affinity receptor for NGF, is expressed on CCs in antral follicles

**Fig. 2:**
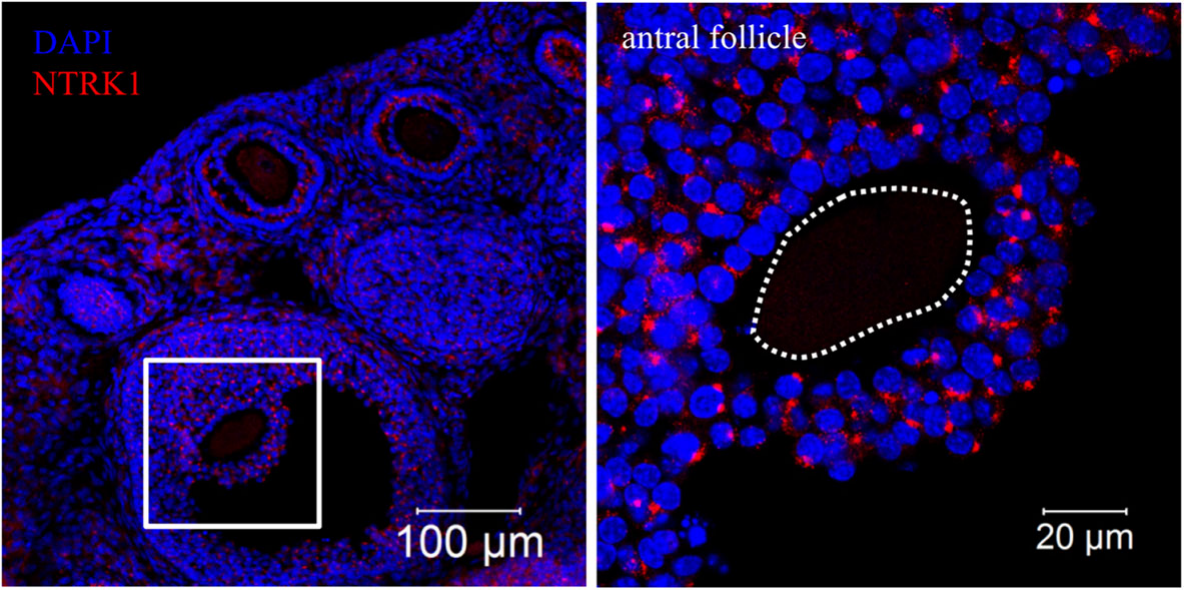
Cellular localization of NTRK1 at the antral follicle stage. Left, representative overview confocal image of NTRK1 staining. Right, magnified image of an antral follicle. Dotted line shows the position of the oocyte. Representative images of three independent experiments are shown

To explore the mechanisms underlying the inhibitory effect of NGF, we analyzed the cellular expression of NTRK1, the high affinity receptor for NGF previously known as TrkA [1]. Intense expression of NTRK1 was detected on the surface of CCs in antral follicles (Fig. 2). Notably, expression of NTRK1 was not detected on oocytes in antral follicles (Fig. 2). Thus, in our in vitro cultured COCs, NTRK1 was exclusively expressed by CCs. To validate the role of NTRK1, we utilized K252α, the phosphorylation inhibitor of NTRK1, during in vitro culture of COCs. The addition of K252α into the culture medium of COCs abolished the suppressive effect of NGF, indicating that the inhibitory effect was mediated by NTRK1. Collectively, these results indicate that excessive NGF inhibit the meiotic maturation of oocytes in COCs via its high affinity receptor NTRK1 which is expressed on CCs.

### The glycolysis of CCs from COCs is down-regulated in the presence of excessive NGF in both mice and humans

**Fig. 3:**
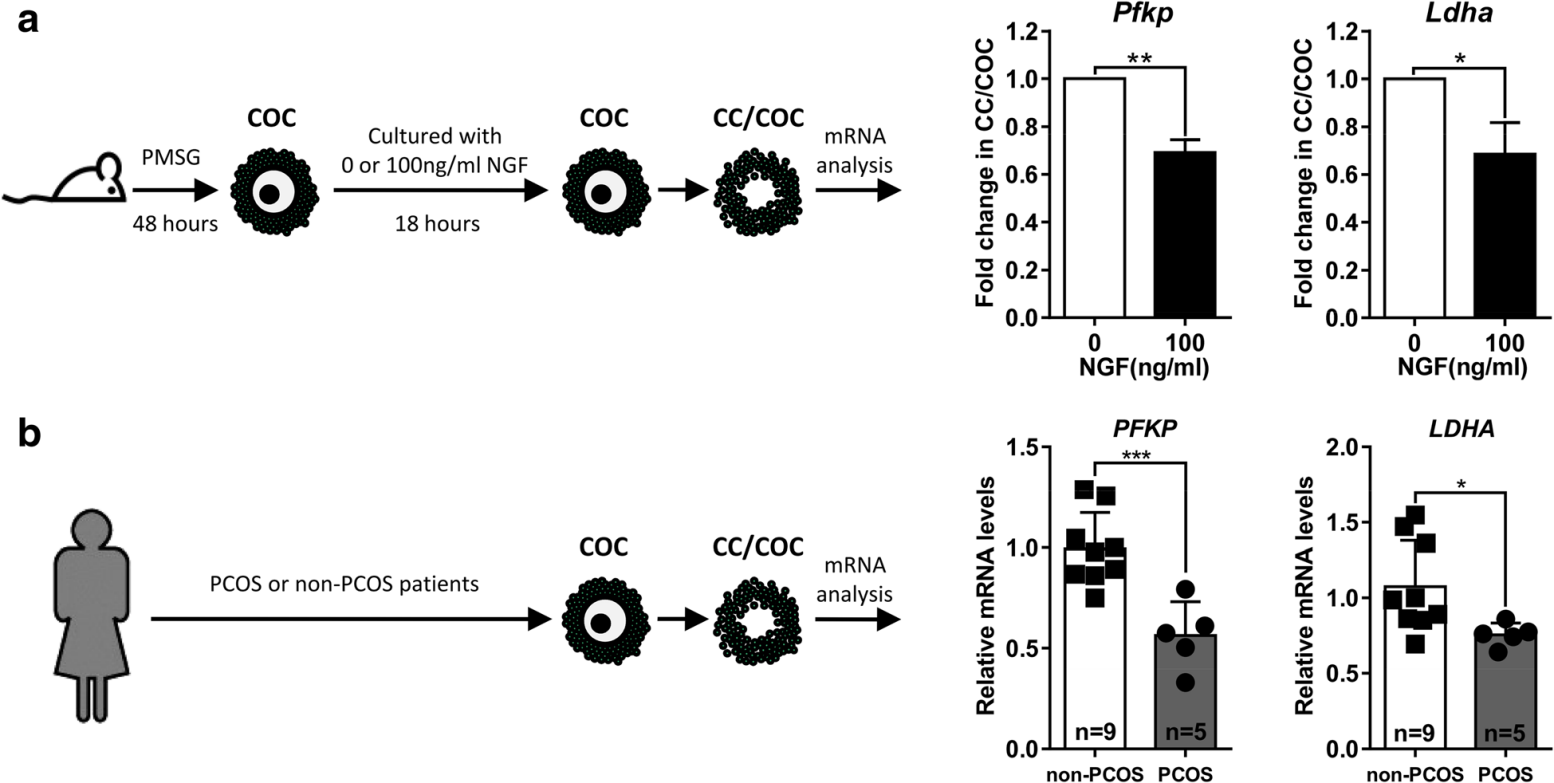
Down-regulation of *Pfkp* and *Ldha* in CCs/COCs occurred in both mice and humans. **a** Relative mRNA levels of *Pfkp* and *Ldha* in mouse CCs/COCs were determined by real-time qPCR, after culturing with 100 ng/ml NGF for 18 h. ≥35 COCs in each group were used and experiments were repeated three times. **b** Relative mRNA levels of *PFKP* and *LDHA* in CCs from non-PCOS (*n* = 9) patients and PCOS patients (*n* = 5) were determined by real-time qPCR. Data are presented as mean ± SEM. The student’s *t*-test was used for statistical analysis. *, *p* < 0.05; **, *p* < 0.01; ***, *p* < 0.001

Because the inhibitory effect of excessive NGF was mediated by NTRK1 expressed on CCs and because glycolysis in the CCs produces the pyruvate that is essential for oocyte maturation, we next analyzed the effect of NGF on glycolysis in CCs. Mouse antral follicle-derived COCs were stimulated by excessive NGF and then CCs were collected to examine the expression of *Pfkp* and *Ldha*, genes encoding rate-limiting enzymes of glycolysis [31]; this allowed us to evaluate the process of glycolysis. The mRNA expression levels of *Pfkp* and *Ldha* decreased significantly in the presence of excessive NGF (Fig. 3a). Similar to the results in mice, the mRNA expression levels of *PFKP* and *LDHA* in CCs from PCOS patients were lower than that in non-PCOS patients (Fig. 3b). *PFKP* was down-regulated by 2-fold (Fig. 3b, left) while *LDHA* was reduced by 1.5-fold (Fig. 3b, right). Collectively, this data suggests that in the presence of excessive NGF, the expression levels of glycolytic rate-limiting enzymes in the CCs of COCs were down-regulated in both mice and humans.

### NGF-induced down-regulation of glycolysis in CCs is dependent on oocytes

**Fig. 4:**
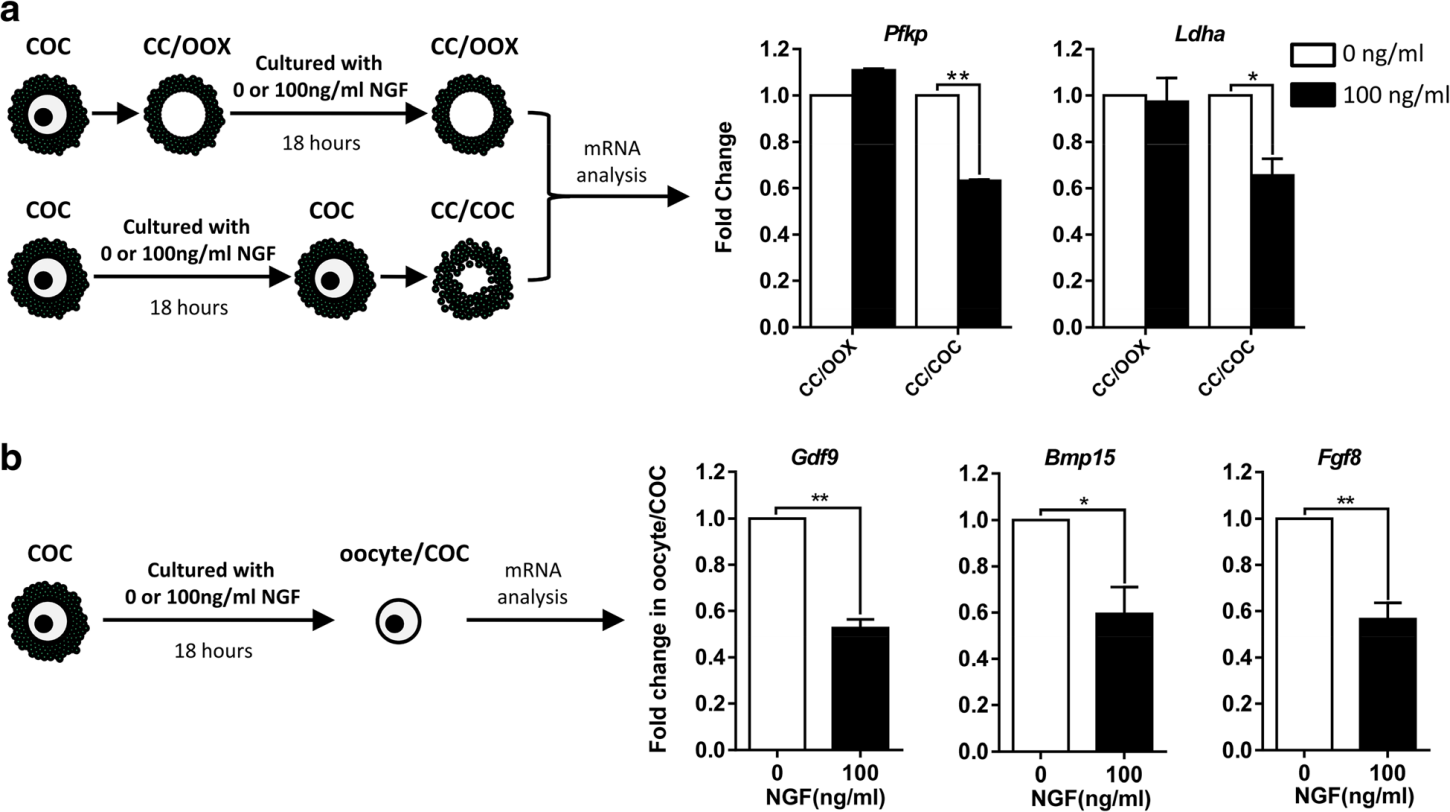
Down-regulation of *Pfkp* and *Ldha* were dependent on oocytes. **a** Relative mRNA levels of *Pfkp* and *Ldha* in CCs from OOX-cumulus cells or CCs/COCs were determined by real-time qPCR in the presence or absence of NGF. CC/OOX, OOX-cumulus cells group; CCs/COC, cumulus cells from the COC group. **b** Relative mRNA levels of *Gdf9*, *Bmp15* and *Fgf8* in oocytes/COCs cultured with or without 100 ng/ml NGF for 18 h were determined by real-time qPCR. ≥35 samples were used in each group. Data of three independent experiments are presented as mean ± SEM. The student’s *t-*test was used for statistical analysis *, *p* < 0.05; **, *p* < 0.01

To observe the direct effect of excessive NGF on CCs, OOX-cumulus cells were treated with excessive NGF but no significant change of mRNA levels of *Pfkp* and *Ldha* was observed (Fig. 4a). Taking into account the significant inhibition of NGF on glycolysis in CCs from cultured COCs (Fig. 4b), it can be concluded that the inhibitory effect of NGF on glycolysis in CCs was dependent on oocytes. Consistent with the down-regulated glycolysis in CCs, the mRNA expression levels of *Gdf9*, *Bmp15* and *Fgf8*, which are three well-characterized ODPFs, were decreased in oocytes from COCs cultured with excessive levels of NGF (Fig. 4b). Levels of *Gdf9* and *Fgf8* were reduced by 2-fold and *Bmp15* was reduced by 1.5-fold. Therefore, the reduced production of ODPFs in oocytes might be responsible for the down-regulation of glycolysis in CCs.

### NGF-stimulated CCs inhibited the production of ODPFs in oocytes

**Fig. 5:**
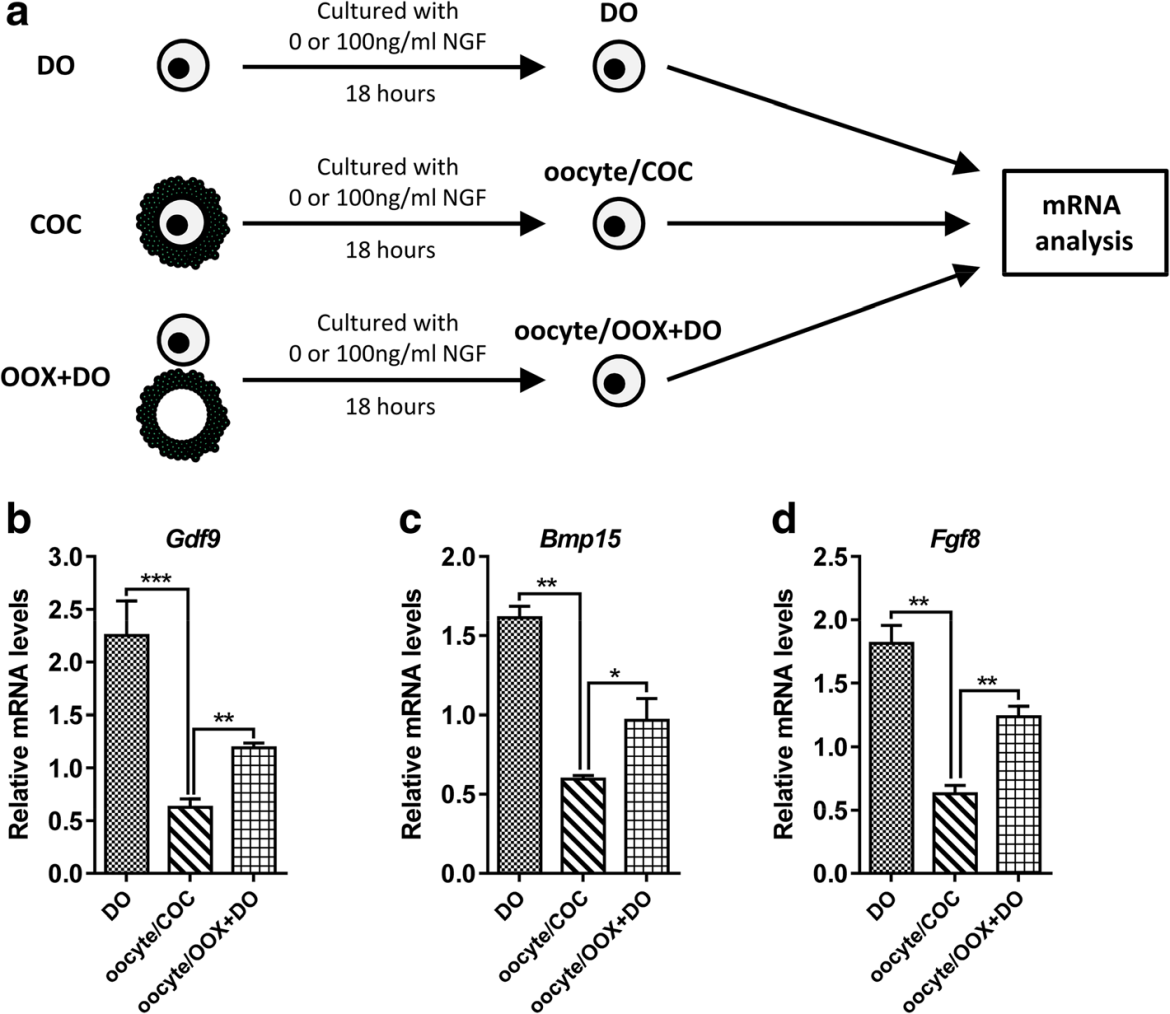
Decrease of ODPFs in oocytes was mediated by CCs. **a** Schematic diagram of the culturing system. **b–d** The relative mRNA levels of *Gdf9* (**b**), *Bmp15* (**c**) and *Fgf8* (**d**) in oocytes respectively from DOs, COCs and “OOX + DO” group were determined by real-time q-PCR cultured in medium with 100 ng/ml NGF for 18 h. DO, denuded oocyte; oocyte/COC, oocytes from COC group; oocyte/OOX + DO, oocytes from the co-cultured OOX-cumulus cells and denuded oocytes group. ≥35 oocytes were used for RNA extraction in each group. Experiments were repeated at least three times independently. Data are presented as mean ± SEM. Student’s two-tailed t-test was used for statistical analysis *, *p* < 0.05; **, *p* < 0.01, ***, *p* < 0.001

**Fig. 6:**
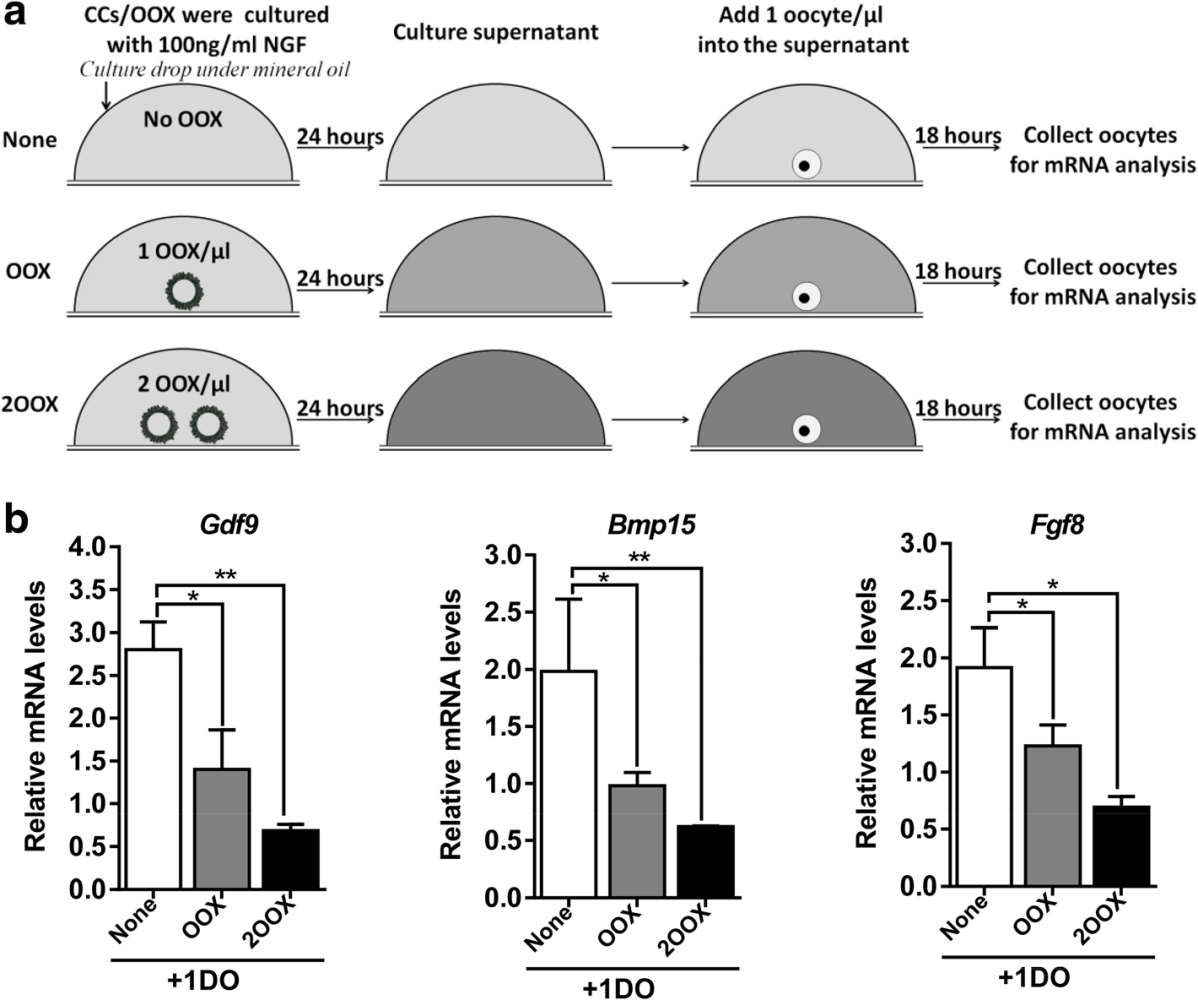
NGF-stimulated CCs inhibited the production of ODPFs in oocytes. **a** Schematic diagram of the culturing system. **b** Relative mRNA levels of *Gdf9*, *Bmp15* and *Fgf8* in oocytes cultured as in (**a**) were determined by real-time qPCR. ≥35 oocytes were obtained for RNA extraction in each group. Data of three independent experiments are presented as mean ± SEM. The student’s *t-*test was used for statistical analysis. *p < 0.05; **p < 0.01

We further explored how excessive NGF caused a reduced production of ODPFs. As NTRK1 is expressed on CCs, but not on oocytes, the reduction in ODPF production in oocytes might be caused by NGF-stimulated CCs. Indeed, when stimulated with excessive NGF, oocytes cultured in the presence of CCs, either in the form of COCs or separately added OOX-cumulus cells to cultured denuded oocytes (referred as “OOX + DO”), expressed less ODPFs than denuded oocytes cultured alone (Fig. 5). These results also implied that the inhibitory effect upon the production of ODPFs was mediated by soluble factors derived from CCs. To validate this, OOX-cumulus cells were stimulated with excessive NGF for 18 h, and the culture supernatant was collected to culture denuded oocytes (Fig. 6a). The expression of ODPFs in denuded oocytes cultured in the supernatant of NGF-stimulated OOX-cumulus cells decreased significantly (Fig. 6b). The inhibitory effect of the supernatant was correlated with the concentration of cultured OOX-cumulus cells. The supernatant of OOX-cumulus cells cultured at a concentration of 2 OOXs/μl exhibited a stronger inhibitory effect than that cultured at a concentration of 1 OOX/μl. Overall, CCs stimulated by excessive NGF inhibited the production of ODPFs in oocytes.

## Discussion

Previous studies have reported that NGF content is significantly increased in the ovarian follicular fluid of PCOS patients from Germany, Italy and Canada [13, 15, 17]. In the present study, we also observed excessive levels of NGF in the follicular fluid of Chinese PCOS patients. The increase of NGF in follicular fluid is associated with lower ovarian responses in aged women [39]. Studies in the rat have shown that the abnormally elevated production of NGF within the ovary increases androgen secretion, disrupts the estrous cycle and is sufficient to initiate several of the structural and functional alterations associated with the development of follicular cysts [40]. In the present study, by treating in vitro cultured COCs with excessive concentrations of NGF, we revealed an inhibitory role of excessive NGF on oocyte maturation. Furthermore, by using well-established models of OOX-cumulus cells, we proved that bidirectional communication between oocytes and CCs mediated the inhibitory function of NGF. Thus, excessive NGF inhibited oocyte maturation in a bidirectional communication dependent manner, indicating a potential pathogenic role of NGF in the progression of PCOS.

According to previous reports, the concentration of NGF in the follicular fluid of PCOS patients varies over a large range across different studies [13, 15, 17]. For example, Dissen et al. reported that the median concentration of NGF in 11 PCOS patients was 5–7 ng/ml [13]. Sadeu et al. further reported that NGF concentration in 16 PCOS patients ranged from 58.5 to 375 ng/ml [17]. In another study, Gulino et al. reported that the mean concentration of NGF in 32 PCOS patients was 2023.30 ± 587.09 pg/mL [15]. In our present study, the concentration of NGF in follicular fluid ranged from 18 to 26 ng/ml for the PCOS group. The differences in NGF concentrations across these studies might be caused by a variety of factors, such as geography, population, ethics and hormone levels [16]. Since the follicular fluid provides a micro-environment for oocyte development and maturation, it is valuable to explore the role of excessive NGF on oocyte maturation. In the present study, we cultured mouse COCs with different NGF concentrations, ranging from 1 to 1000 ng/ml, which covered the wide range of NGF concentrations reported in previous studies. Combining NGF concentration in the follicular fluid of PCOS patients and the effect of different NGF concentrations during in-vitro culturing, 100 ng/ml of NGF was considered as a moderate concentration to mimic excessive NGF because of its significant inhibitory effect and was therefore used for subsequent experiments throughout the present study.

To explore the mechanisms underlying the inhibitory effect of NGF, we analyzed the cellular expression of NGF receptors. Two different NGF receptors have been characterized: NTRK1, the high affinity receptor for NGF and previously known as TrkA; and p75, a pan-specific receptor for NT family members [1]. NTRK1 is expressed on mesenchymal cells, CCs and granulosa cells in mouse antral follicles, while p75 is only expressed on mesenchymal cells [1, 4]. Consistent with these previous reports, we detected NTRK1 expression on CCs but not on oocytes in mouse antral follicles. Thus, in our in vitro culturing system, CCs were the cells directly affected by excessive NGF. Moreover, the application of K252α abolished the inhibitory effect of excessive NGF, further validating the involvement of NTRK1. In human antral follicles, the expression of NTRK1 on CCs has been reported by several studies [1, 3], while its expression in oocytes remains controversial. Nevertheless, human CCs are also capable of being stimulated by NGF in the follicular fluid. Besides the uniform expression of NTRK1 on CCs in mice and humans, decreased glycolysis, indicated by the reduced expression of *Pfkp* and *Ldha*, was observed in both mouse CCs from COCs treated with excessive levels of NGF and human CCs from the COCs of PCOS patients. As it is very difficult to obtain human samples for research, their number was low. Be that as it may, these specimens strengthened the hypothesis that was initially observed by us in mice. Since the CCs of PCOS and non-PCOS patients were collected from patients who received ICSI treatment, the corresponding oocytes were used for embryo culturing and transfer, making it impossible to analyze the expression of ODPFs in human oocytes. However, previous reports showed that the expression of GDF9 and BMP15 in oocytes from PCOS patients was reduced compared to normal ovulatory women [41, 42]. Collectively, these results suggested that the mechanisms we found in mice were also applicable for PCOS patients.

Excessive NGF inhibited the meiotic maturation of COCs cultured in vitro, suggesting that NGF can contribute to the pathogenesis of PCOS by impairing the development of COCs. Although we proved that bidirectional communication between CCs and oocytes was necessary for the inhibitory effect of excessive NGF, further experiments need to be conducted in the future. Initially, experiments treating CC/COC with GDF9, BMP15 and FGF8 in the presence of NGF should be conducted to demonstrate that ODPFs can reverse the suppression of *Pfkp* and *Ldha* by excessive NGF. Secondly, how NGF-stimulated CCs down-regulate the production of ODPFs in oocytes is still unclear. Our results suggested the existence of CC-derived inhibitory factor(s). Further identification of these inhibitory factor(s) is therefore very valuable. On the other hand, in the antral follicles of both mice and humans, mesenchymal cells are also reported to express NGF receptors [1]. Thus, mesenchymal cells are also potential target cells for excessive NGF in PCOS patients. Overall, it is valuable to clarify the pathogenic roles of excessive NGF and therapies targeting NGF may be beneficial for patients suffering from PCOS.

## Conclusions

**Fig. 7:**
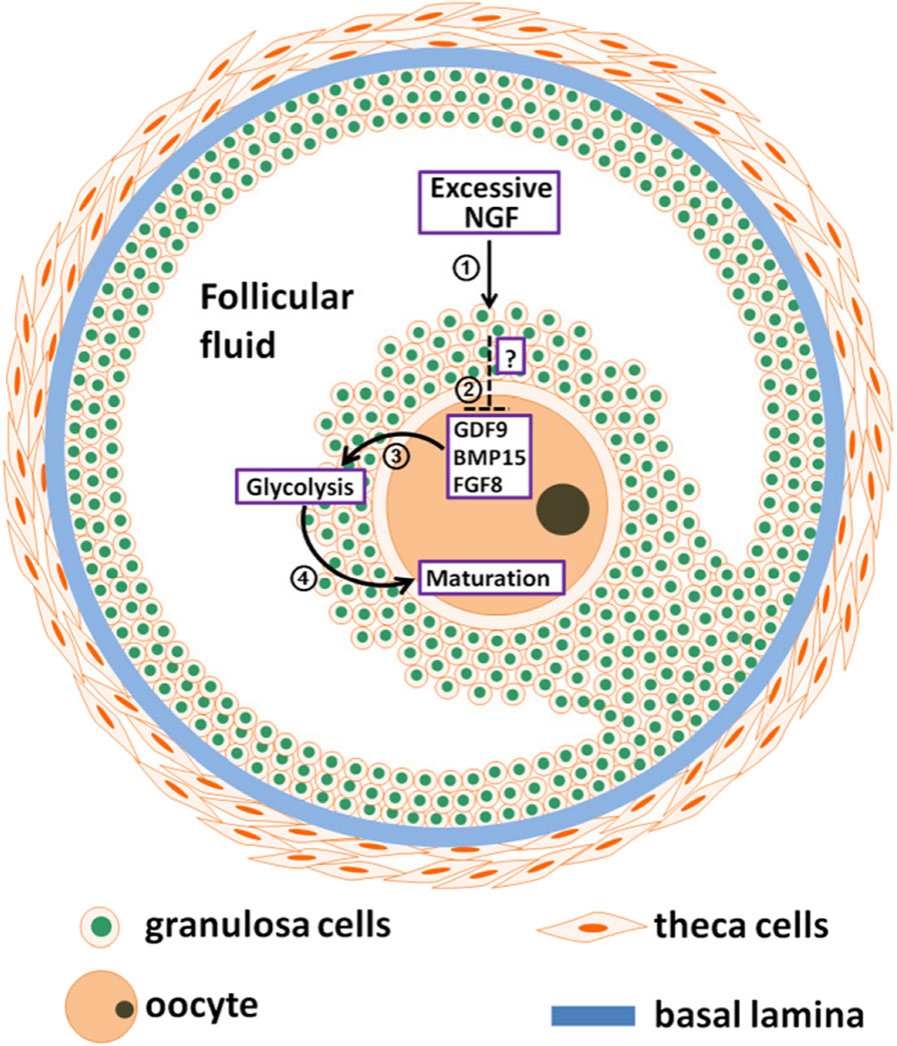
Working model. ① Excessive NGF in the follicular fluid of PCOS patients. ② Stimulated-CCs secreted inhibitory factor(s) (undefined yet) and down-regulated the production of ODPFs in oocytes. ③ Down-regulation of ODPFs could reduce glycolysis in CCs. ④ Reduction of glycolysis in CCs impaired oocyte meiotic maturation at the antral follicle stage

The present study could be summarized as Fig. 7. It demonstrates that in vitro addition of excessive NGF inhibits glycolysis in CCs and maturation of oocytes in mouse COCs and the inhibition is dependent on bidirectional communication between oocytes and CCs. Pharmacological blocking of NGF-high affinity receptor NTRK1 by K252α abolished the inhibitory effect on oocyte maturation. Excessive NGF inhibits glycolysis in CCs of COCs, at least in part, by reducing the production of ODPFs in oocytes. Importantly, dysfunction of glycolysis in CCs was identified in PCOS patients with excessive NGF in the follicular fluid. Our findings revealed a pathogenic role of excessive NGF in PCOS.

## Abbreviations

BMP15: Bone morphogenetic protein 15
BSA: Bovine serum albumin
CCs: Cumulus cells
COC: Cumulus-oocyte complex
ELISA: Enzyme linked immunosorbent assay
FGF: Fibroblast growth factor
FGF8: Fibroblast growth factor 8
GDF9: Growth differentiational factors 9
ICSI: Intracytoplasmic sperm injection
IVF: In vitro fertilization
LDHA: Lactate dehydrogenase A
NGF: Nerve growth factor
NTRK1: Neurotrophic receptor tyrosine kinase 1
ODPFs: Oocyte-derived paracrine factors
OOX-cumulus cells: Oocytectomized cumulus cells
PBS: Phosphate buffered saline
PCOS: Polycystic ovary syndrome
PFKP: Phosphofructokinase, platelet
PMSG: Pregnant mare serum gonadotropin
